# Modeling urban malaria infection in *Anopheles stephensi* hotspot area in Eastern Ethiopia: application of Structural Equation Modeling

**DOI:** 10.1186/s12879-025-11841-2

**Published:** 2025-11-05

**Authors:** Hailu Merga, Teshome Degefa, Zewdie Birhanu, Ephrem Abiy, Ming-Chieh Lee, Guiyun Yan, Delenasaw Yewhalaw

**Affiliations:** 1 Department of Epidemiology, Institute of Health, Jimma University, Jimma, Ethiopia; 2 School of Medical Laboratory Sciences, Institute of Health, Jimma University, Jimma, Ethiopia; 3 Tropical and Infectious Diseases Research Center, Jimma University, Jimma, Ethiopia; 4 Departement of Health, Behavior, and Society, Faculty of Public Health, Jimma University, Jimma, Ethiopia; 5 Abt Global PMI Evolve Project, Addis Ababa, Ethiopia; 6 Program in Public Health, University of California at Irvine, Irvine, USA

**Keywords:** Urban malaria, Structural equation modeling, Malaria infection, Ethiopia

## Abstract

**Background:**

In Ethiopia, the fight against malaria faces significant challenges, including the emergence of insecticide resistance, vector behavioral change, population movement, climate change, civil unrest, emergence of COVID-19 pandemic, unplanned urbanization, invasion and spread of urban malaria vector *Anopheles stephensi*. Modeling the complex relationship and contribution of these factors to malaria infection is essential for ultimate malaria elimination. Hence, the aim of this study is to model the direct and indirect effect of factors affecting the risk of urban malaria infection in eastern Ethiopia where an invasive malaria vector has been recently detected.

**Methods:**

A facility based cross-sectional study was conducted among 329 febrile urban resident patients visiting public health facilities of Dire Dawa city using an interviewer administered questionnaire. Structural Equation Modeling (SEM) was done to identify the direct and indirect effects of factors for malaria infection. Lavaan (Latent variable analysis) package was used in R and diagonally weighted least square (DWLS) estimation method was employed.

**Results:**

The confirmatory factor analysis indicated that all selected factors were significantly loaded on their respective latent variables. The direct effect of the final model indicated that wealth index had a negative statistically significant effect on insecticide treated nets (ITN) utilization (-0.66; *p* < 0.001) and knowledge on malaria and its prevention (-0.63; *p* < 0.001). Attitude had positive effect on ITN utilization (0.16; *p* = 0.049) and having history of travel outside the city had significant positive effect on malaria infection (0.969; *p* = 0.01). The indirect effect analysis revealed two pathways in which attitude and utilization as the mediating factor significantly influenced the risk of malaria infection (indirect path coefficient=-0.091; *p* = 0.038) and (indirect path coefficient = 0.029; *p* = 0.048) respectively.

**Conclusion:**

SEM is an effective technique that identified the direct and indirect effects of wealth index, ITN utilization, knowledge, attitude and history of travel on risk of urban malaria infection. Hence, strengthening holistic approach and urban-targeted malaria interventions should be enhanced to prevent and control malaria infection in urban settings.

**Supplementary Information:**

The online version contains supplementary material available at 10.1186/s12879-025-11841-2.

## Background

Despite significant progress made in reducing malaria morbidity and mortality, the disease remains a severe worldwide public health concern. According to the World Health Organization (WHO) world malaria report 2023, there were 249 million malaria cases in 85 endemic countries and territories, an increase by 5 million from 2021. About 94% of these cases came from countries in the WHO African region [1].

Malaria has been overlooked in urban settings for centuries, but recently, WHO and the United Nations developed a global framework for malaria response in urban settings [2]. Despite increasing urbanization, many African countries continue to suffer from sub-standard housing, unplanned urban expansion, inadequate sanitation, and poor surface water drainage, all of which would provide an ideal condition for mosquito proliferation. Studies suggest that urbanization has a significant epidemiological, entomological, parasitological, and behavioral implications on malaria risks [3–9]. Besides, the spread of the invasive urban malaria vector, *An. stephensi*, poses a major challenge to control in urban areas [10–13].

On the other hand, population movement has the ability to alter malaria transmission patterns by introducing the parasite to new areas and affecting local immunity and transmission dynamics. Furthermore, without access to health care and prevention, travelers are more prone to malaria infection and contribute more to transmission [14–17]. Studies from Malawi and Uganda indicated that malaria in urban areas were strongly associated with travel history [18, 19]. Furthermore, the WHO 2023 report indicated that the percentage of the population sleeping under an ITN increased between 2000 and 2022 for the whole population from 2% to 49%, which indicated that yet there is poor utilization [1]. Similarly, a recent finding from southwestern Ethiopia showed 37.9% ITN utilization and traveling history as well as history of malaria illness as risk factors for malaria [20]. A systematic review done on malaria and urbanization focusing on sub-Saharan Africa showed that artificial urban vector breeding sites, urban agriculture, and low socioeconomic status as a significant risk factors for urban malaria [9]. Knowledge and attitude of malaria and its prevention are also one of the main factors in malaria prevention and control [21–23]. Recently, WHO declared COVID-19 an established and ongoing health issue that no longer constitutes a public health emergency of international concern. However, COVID-19 can play a significant role in malaria elimination efforts and distribution of malaria prevention and control resources [1, 24].

The introduction and spread of the urban malaria vector, *An. stephensi*, into African countries mainly Ethiopia, Sudan, Somalia and Djibouti exacerbates the already complex malaria landscape, with projections indicating that it could place an additional 126 million Africans at risk of contracting malaria and lead to a 50% increase in malaria cases in Ethiopia [10, 25]. This vector adapts to its environment, breeds in man-made water containers and exhibits resistance to several insecticide classes. If left uncontrolled, its spread across the Horn of Africa, coupled with rapid and poorly planned urbanization, could escalate the risk of malaria outbreaks in African cities [6, 10, 11, 13, 26–28]. In Ethiopia, the emergence of insecticide resistance, population movement, climate, civil unrest, that disrupt malaria prevention and treatment campaigns, unplanned urbanization, emergence and spread of this vector are the major challenges in the fight against malaria [29].

Though there are many studies done to identify risk factors of malaria infection in Ethiopia, there is no single study that modeled together the direct and indirect effect of the risk factors of malaria infection in urban settings. Therefore, this study hypothesized to model the direct and indirect effect of socio-demographic index, wealth index, environmental factors, knowledge related factors, attitude related factors, ITN utilization related factors, history of malaria diagnosis, history of travel outside the study area, and COVID-19 test and vaccination related factors for urban malaria infection in eastern Ethiopia using Structural Equation Modeling technique (SEM). Better understanding of the effect, strength, and direction of relationships among these factors will help to identify precise areas for targeted interventions to most effectively eliminate *An. stephensi*.

In this study, the SEM- approach was employed because it is a modern powerful multivariate statistical method that allows the evaluation of a series of variables for representing, testing and estimating the complex relationships among variables (measured variables and latent constructs) [30–36]. Furthermore, SEM is an analytical approach that can be used to model risk of infectious diseases infection concepts that cannot readily be addressed in traditional tests of association, such as regression analyses [30, 33, 36, 37]. As far as we know, this is the first study that aimed to establish the SEM to explore the urban malaria risk factors in Ethiopia.

## Methods

### Study design and setting

**Fig. 1:**
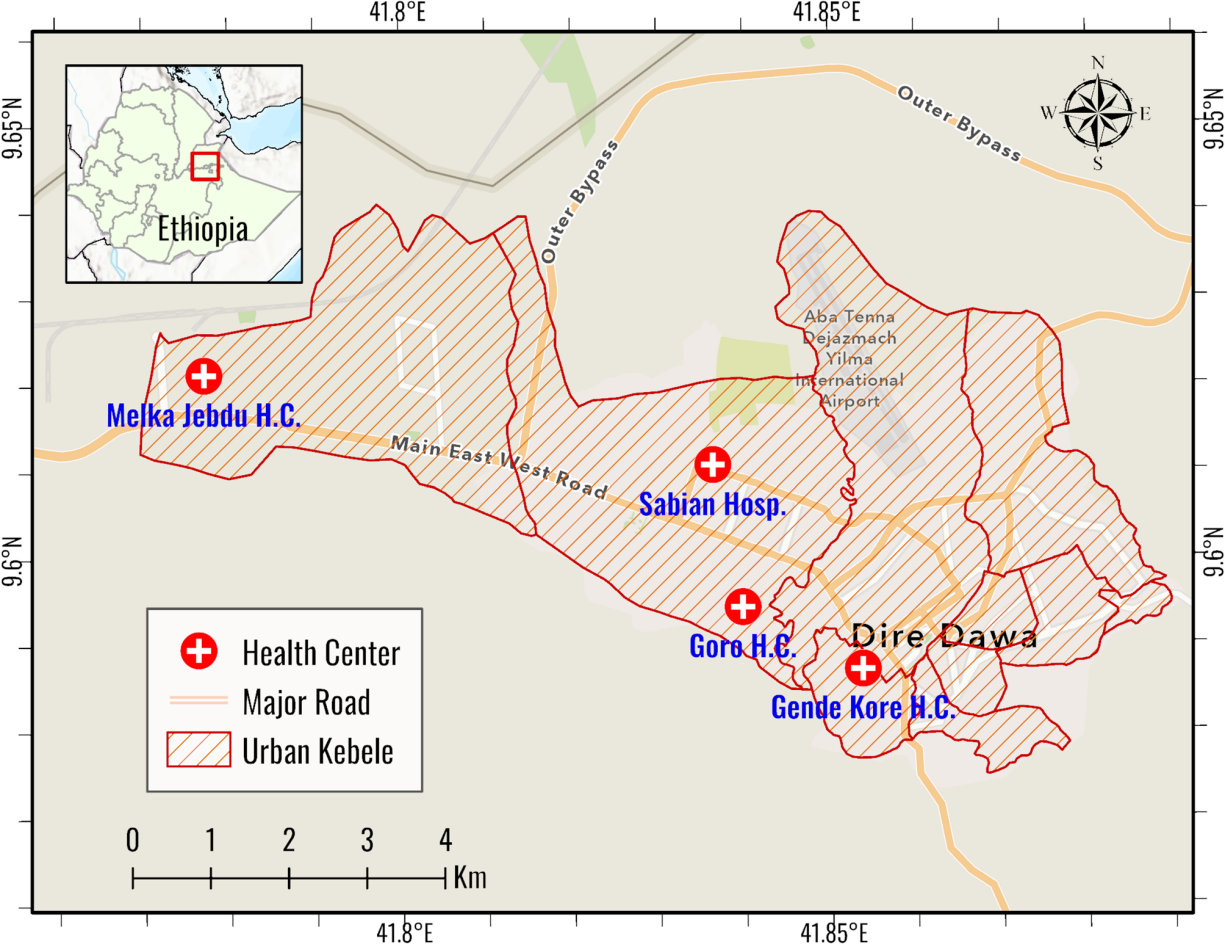
Map of the study area (Dire Dawa city, 2023)

The study was conducted between May 7, 2023 and May 30, 2023 in Dire Dawa city. It is located 515 km southeast of Ethiopia’s capital, Addis Ababa, and 311 km west of Djibouti and is a vital logistics hub for goods and cargo transportation. The city administration had total population of 445,050, about 74% reside in urban areas. It is one of the two city administrations in the Federal Democratic Republic of Ethiopia and located between 90 28.1” N and 90 49.1” N latitude and between 410 38.1” E and 420 19.1” E longitude. The city experiences a warm, dry climate with limited rainfall, averaging 624 mm annually, and temperatures ranging from 19 °C to 32 °C throughout the year. In Dire Dawa, malaria incidence has historically been low, with an annual parasite clinical incidence of less than 5 cases per 1,000 people between 2014 and 2019. However, recently, the city has experienced a sharp rise in malaria cases, mainly driven by the rapid spread of the invasive *An. stephensi* mosquito vector. The outbreak reflects a growing trend of urban malaria across Africa, posing a serious public health threat​. Currently, there is vector surveillance and ongoing vector control interventions like larval source management. There are two public hospitals (Dilchora Referral Hospital and Sabian Primary Hospital), six private hospitals (1 General and 5 Primary Hospitals), 10 Health Centers and 59 Private Clinics in the city (Fig. 1).

### Population and sampling

All selected febrile patients who visited the public health facilities were the source population. Febrile patients who were unable to respond or had communication problems, or unwilling to participate in the study and those who were from rural areas were excluded from this study.

The sample size for this study was determined using Daniel Sope`s online Sample Size calculation tool for Structural Equation Modeling with the following assumptions: number of observed and latent variables in the model, the anticipated effect size, and the desired probability and statistical power levels. Accordingly, 0.3 anticipated effect size, 0.8 desired statistical power, 9 latent variables and 59 observable variables and 0.05 probability level were used in this study. Finally with the addition of 10% non-response rate, a total of 329 the sample size was used for this study. Assuming that all the health facilities were likely to be homogeneous in receiving clients, proportional allocation was done based on the average of the previous six-moths report of febrile cases.

### Data collection tool and procedure

Data was collected using an interviewer administered structured questionnaire developed from different relevant and related literatures [3, 4, 7, 9, 18–20, 38–45]. Variables used for household wealth index construction were developed based on literature and the Ethiopian Demographic and Health Survey [46]. The questionnaire was written in English language and translated to both Afan Oromo and Amharic languages, and after completion, they were translated back to English language to check for consistency. Data were collected by trained and experienced data collectors and laboratory technicians who were assigned to the health facilities until the sample size for each health facility was attained. A capillary blood sample was collected from each study participant following standard operation procedures. Finally, the slides were stained with 10% Giemsa for 10 min, air dried, and examined microscopically by an experienced laboratory technologist. The slides were considered negative if no malaria parasite was seen after examining 100 high power fields.

To ensure the quality of data, a pretest was done among 5% of the sample size at Goro Health center in Dire Dawa city. Training for data collectors (interviewers, laboratory technologists and supervisors) was given on how to collect data using ODK (Open Data Kit) software, data collection approach and how to handle the sample as well as how to conduct laboratory investigations using standard operating procedures. As part of quality control, all malaria positive blood smear slides and 10% of the negative slides were re-examined by another blinded senior laboratory technologist.

### Hypothesized model building and measurement variables

Hypothesizing a model in SEM involves specifying both the measurement and structural components based on theoretical considerations and empirical evidence. In this hypothesized model, we aimed to identify the direct and indirect effect of Socio-demographic index (SDIndex), wealth index (WI), knowledge about malaria infection and its prevention (K), attitude towards malaria infection and its prevention (AT), ITN utilization (UT), COVID-19 test and vaccination impact (COV), environmental factors (E), history of malaria infection (Dx) and history of travel outside the study area (TR). In the measurement model, we defined the latent variables and their corresponding observed indicators. Wealth index was measured by eleven indicators (WI1 to WI11), knowledge (K) was measured by nine indicators (K1 to and K9), attitude (AT) was measured by thirteen indicators (AT0 to AT12), ITN utilization (UT) was measured by five indicators (UT1 to and UT5), COVID-19 Impact (COV) was measured by three indicators (Cov1 to Cov3), environmental factors (E) was measured by eight indicators (E1 to E8), previous history of malaria infection (Dx) was assessed by three indicators (Dx1 to Dx3) and history of travel outside the city (TR) was measured by three indicators (TR1 to TR3). We hypothesized that all these latent variables have direct effect on malaria infection. Except attitude (measured with Likert scale) and sociodemographic index variables, the response options of all those variables were “yes” and “No” **(S1).**

Accordingly, the structural model hypothesizes the relationships among these latent variables. Specifically: Attitude (AT) is predicted by ITN utilization (UT), knowledge (K) is predicted by wealth index (WI) and Attitude (AT) is predicted by wealth index (WI) and Knowledge (K). Moreover, ITN utilization (UT) is predicted by wealth index (WI), knowledge (K), and attitude (AT). We aimed also to specify the covariances between certain latent variables to account for their potential correlations. Hence, we aimed to assess the covariance between wealth index (WI) and knowledge (K), attitude (AT) and knowledge (K), and the covaries between ITN utilization (UT) and travel history (TR).

Finally, we hypothesized to assess the indirect effects to understand how wealth index (WI) influences malaria infection through attitude (AT), knowledge (K) and ITN utilization (UT). Similarly, the indirect effect of attitude (AT) on malaria Infection via utilization (UT) and the indirect effect of knowledge (K) on attitude (AT) and subsequently on malaria Infection was assessed.

### Data processing and analysis

A checklist for reporting model setting and interpretation of results in SEM was used to analyze and report the data [32]. Before further analysis, data cross-verification and data cleaning were done. The R software lavaan package for SEM was used using Diagonally Weighted Least Square Method (DWLS) estimation method. The lavaan package provides tools for checking these assumptions and ensuring the model’s validity and reliability through various diagnostic measures and fit indices. Structural Equation Modeling is a comprehensive and flexible approach that consists of studying the relationships between variables. It is a technique that allows not only to identify the direct and indirect effects between variables, but also to estimate the parameters of varied and complex models. It is different from other type of analysis in that it can manage many variables simultaneously, it has greatest statistical power, and it is causal modeling with caution of result interpretation [33, 37, 47].

Model fitness was checked with key fit indices such as the comparative fit index (CFI), tucker-Lewis Index (TLI), root mean square error of approximation (RMSEA), and standardized root mean square residual (SRMR). Accordingly, ratio of chi-square to the degree of freedom (χ2/df) < 5, CFI *≥* 0.95, TLI *≥* 0.95, RMSEA *≤* 0.08 and SRMR *≤* 0.06 was considered as a good fit [33, 48]. A standardized factor loading greater than 0.5 was used to show the presence of a good relationship between items and respective latent variable. Similarly, Cronbach’s alpha was used to test the consistency of latent constructs measured by observable variables, with a value greater than 0.5 serving as the cutoff point for including each item in the model. The adequacy of sample size was checked using Kaiser–Meyer–Olkin (KMO).

When the number of observable and latent variables is big and the model has a complicated structure, it might be difficult to decide which component to review first for model modification if the model’s fit is poor. A good technique in this scenario would be to separate the measurement model from the structural model and review them one by one [32]. Hence, in the first phase, we focused on the measurement model, employing confirmatory factor analysis (CFA) to validate the instruments and ensure that the observed variables accurately measured the latent constructs. The second phase involved examining the relationships between the latent variables by estimating the standardized path coefficients and their significance levels (p-values) to determine the strength and direction of the hypothesized relationships. Finally, the hypotheses were tested by evaluating the standardized path coefficients and their statistical significance, allowing us to confirm or refute the proposed relationships based on empirical data. Graphs and tables were used to display the results.

## Results

### Socio-demographic characteristics of respondents

A total of 337 febrile patients were approached for data collection. Of these, complete data were obtained from 329 (97.6%) participants. The mean age (*±* SD) of the respondents was 28.2 (+ 14.4). Majority of the respondents 245 (74.5%) were males. Regarding their marital status, more than half of the respondents 178 (54.1%) were single and two third of them 134 (40.73%) were married. Nearly half, 145 (44.1%), of the respondents attended primary school followed by 118 (35.87%) secondary school. With their occupational status, majority of the respondents 107 (32.52%) were traders. The average family size was 4 with the maximum of 11 and minimum of 1 family size (Table 1).

**Table 1:** The sociodemographic characteristics of study participants from dire Dawa, Eastern Ethiopia, 2024

Variable	Variable category	Frequency (*n*)	Percentage (%)
Sex	Male	245	74.47
	Female	84	25.53
Age		Mean (SD) = 28.2 (*±* 14.4)	
Marital status	Single	178	54.10
	Married	134	40.73
	Divorced	10	3.04
	widowed	7	2.13
Educational status	Primary (1–8)	145	44.07
	Secondary (9–12)	118	35.87
	Technical and vocational Training (TVET)	7	2.13
	University degree and above	19	5.78
Occupational status	Trader	107	32.52
	Student	82	24.92
	Factory/Construction worker	48	14.59
	Unemployed	38	11.55
	Employee/Office Worker/Teacher	30	9.12
	Driver	24	7.29
Family size	4 and above	219	66.57
	Less than 4	110	33.43
Presence of < 5 child in the household	Yes	115	34.95
	No	214	65.05

### Reliability and correlation analysis

The reliability of the observed variables was assessed and the variables with value less than 0.5 were excluded from the latent variable formation. The correlation analysis of each observed variable was assessed and there was both positive and negative correlation between those variables. Similarly, the correlation between latent variables was conducted (S2)**.**

### Confirmatory factor analysis and modification indices

Nine latent variables were identified as follows: sociodemographic index, environmental factors, wealth index, knowledge of malaria prevention and control related factors, attitude of malaria and its prevention related factors, ITN utilization related factors, travel history, history of malaria diagnosis and COVID-19 related factors (as mentioned above in detail). The adequacy of the sample size was checked using KMO and its value was 0.5, indicating the appropriateness of data to proceed with confirmatory factor analysis.

**Fig. 2:**
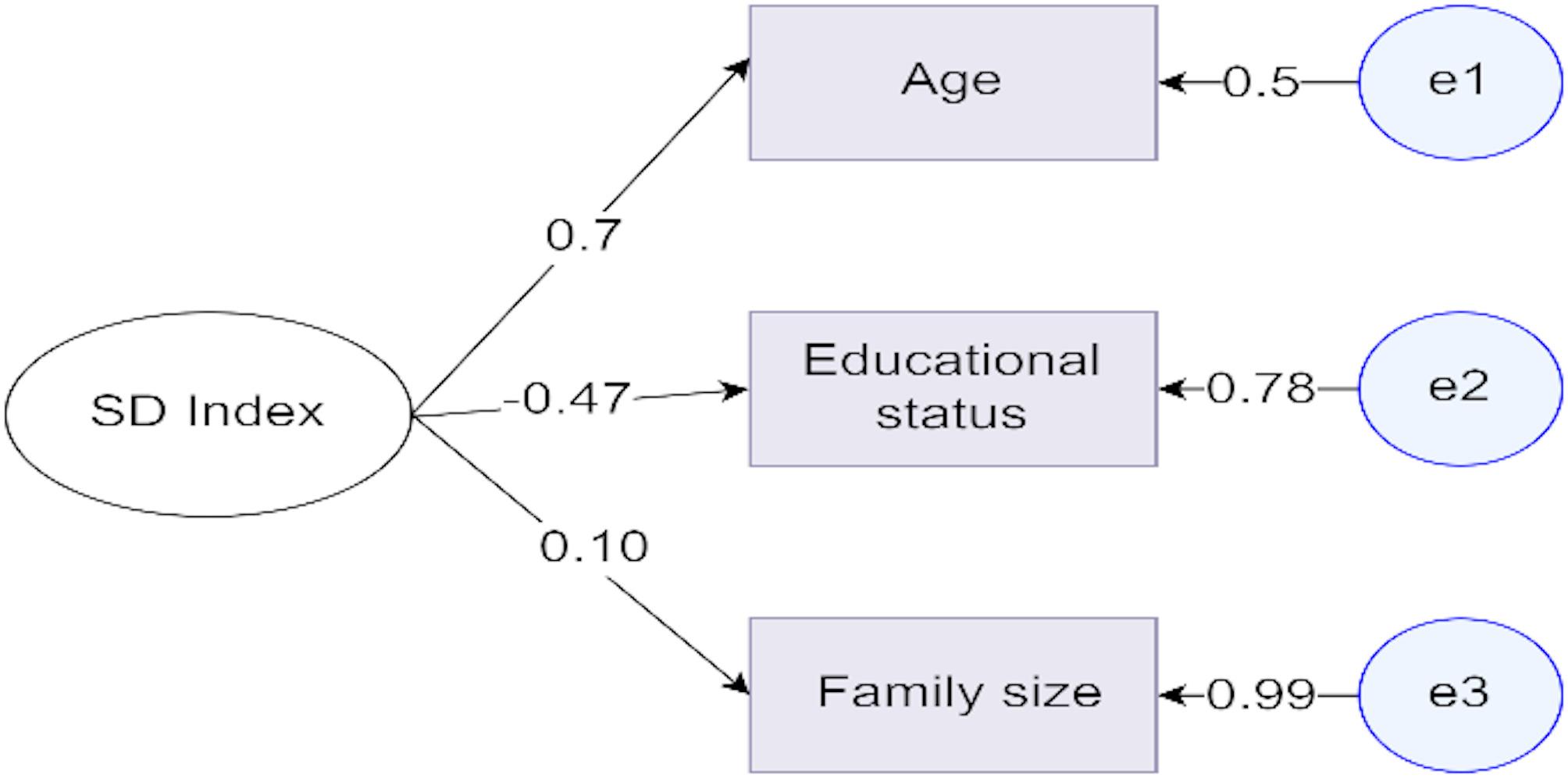
Sociodemographic Index variables factor loadings. Note: oval shape shows the latent variable, rectangle shows the observed variables and the circle shows residual errors. Note: oval shape shows the latent variable, rectangle shows the observed variables and the circle shows residual errors

The factor loading of each latent variable was identified in advance before running the final structural model. Modification indices, valuable tools in SEM of lavaan for identifying potential model improvements, were done to indicate good model fit. It provides specific suggestions for modifying the model to better fit the data. Hence, in this study, each latent variable factor loadings were done separately before running confirmatory factor analysis of all latent variables. Finally, the final model was developed with the identified factor loading values above 0.5. Accordingly, three observed variables (age, educational status, and family size) were used for the sociodemographic index. As we can see from the Fig. 2 below, the factor loadings for the age, educational status and family size are 0.1, −0.47 and 0.70 respectively (Fig. 2).

**Fig. 3:**
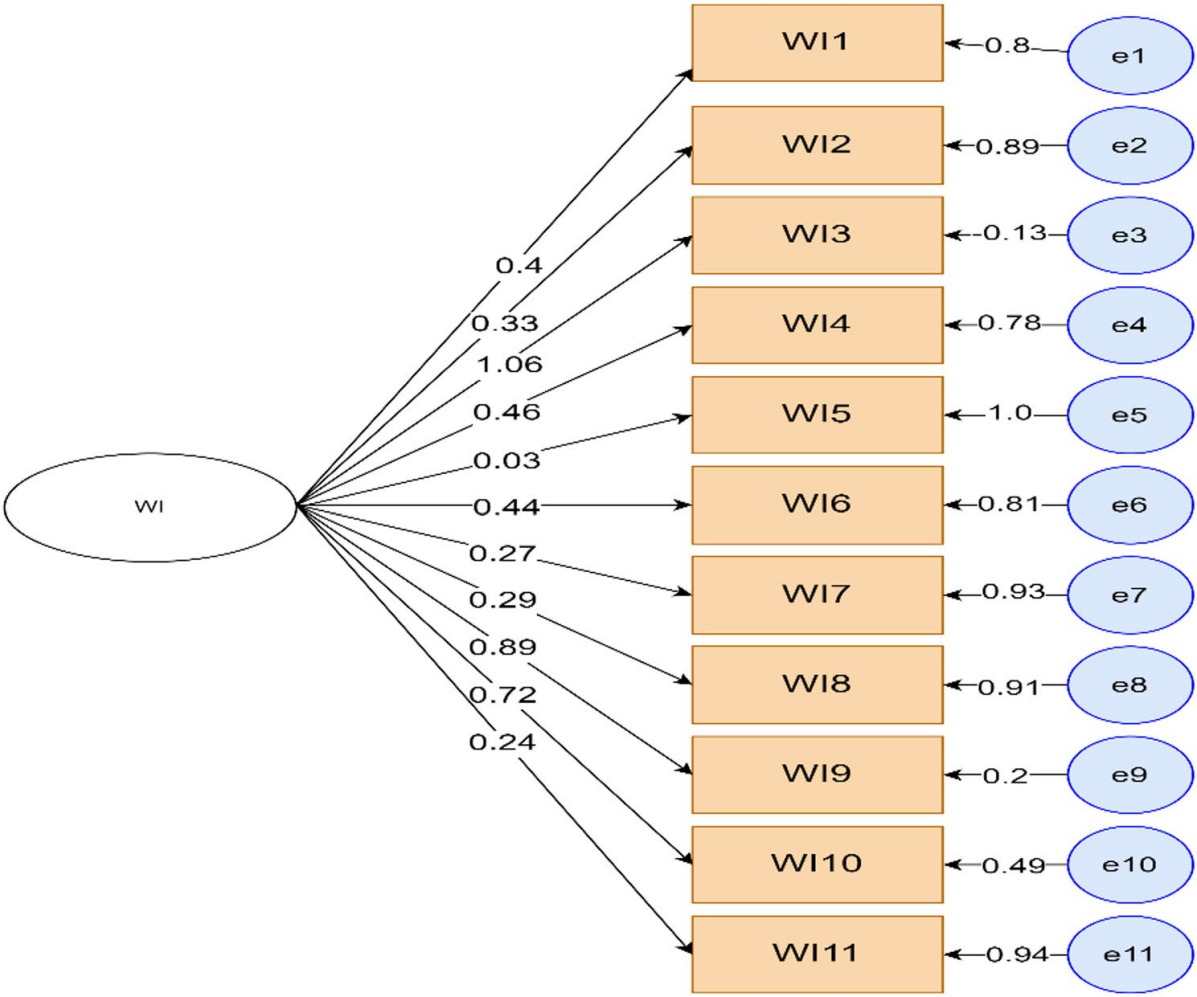
Factor loadings of wealth index observable variables. WI = Wealth Index (WI1 to WI11)

The factor loading of observed variables to measure wealth index was also identified before running the final structural model. Accordingly, eleven observed variables were used for the wealth index and their loading factor is as shown in the Fig. 3 below (Fig. 3).

**Fig. 4:**
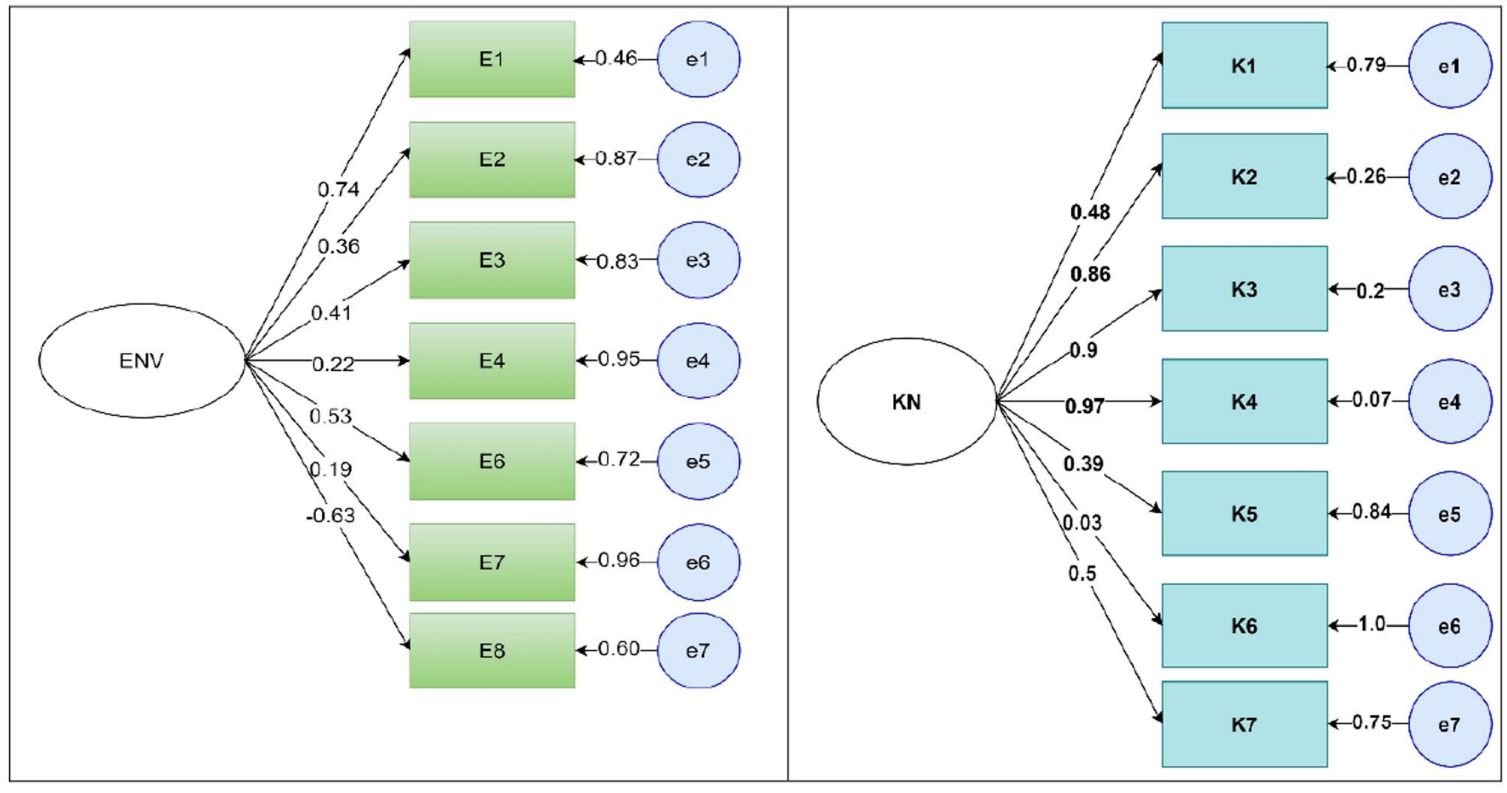
Factor loadings of Environmental factors and knowledge related observable variables. (**a**) E = environmental observable factors, (E1 to E8), (**b**)k = knowledge observable factors, (k1-k7)

The latent variable environmental factors were also assessed with 6 observed variables. Similarly, the knowledge of respondents on malaria and its prevention was also assessed as a latent variable with seven observable variables (Fig. 4).

**Fig. 5:**
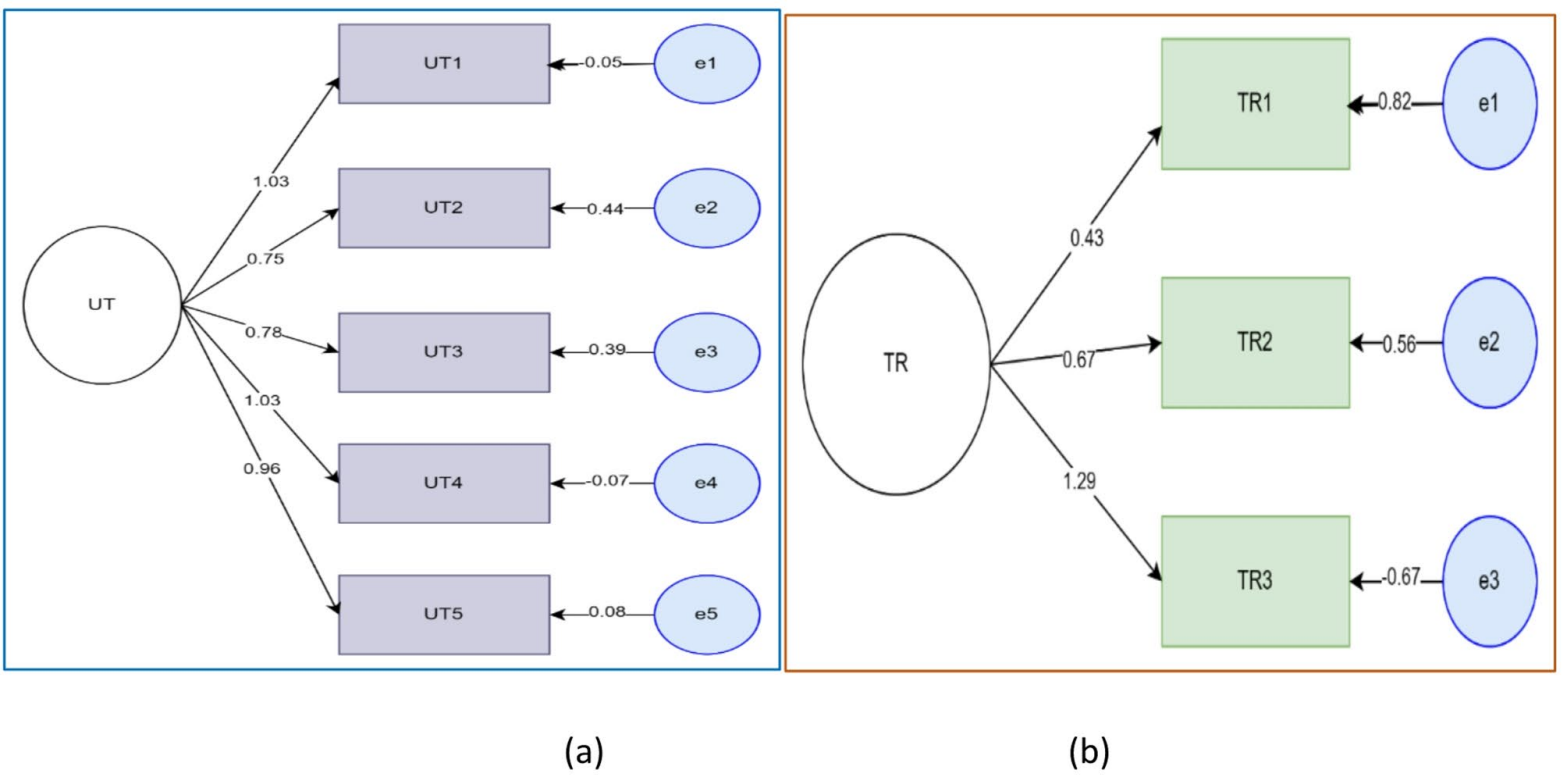
Factor loadings of ITN utilization and travel history related factors observable variables. (**a**) *UT* = Utilization observables,*(UT1- UT5)*,(**b**) TR = Travel history observables(TR1-TR3)

ITN utilization of respondents for urban malaria prevention was assessed as a latent variable with five observable variables. History of travel (within the month of the data collection and before a month) outside of the study area of respondents was also assessed as a latent variable with three observable variables (Fig. 5).

**Fig. 6:**
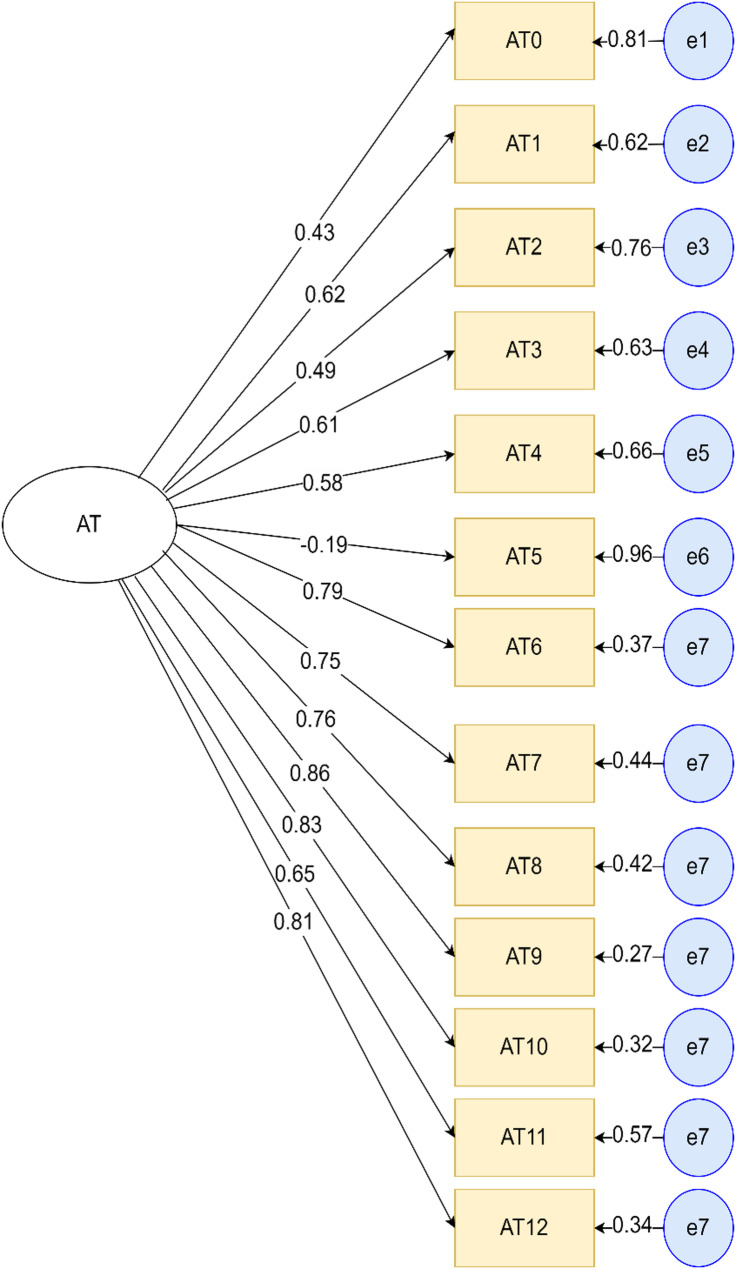
Factor loadings of attitude related factors observable variables. *AT = Attitude (AT0 -AT12)*

Attitude of respondents on malaria and its prevention was also assessed as a latent variable with thirteen observable variables (Fig. 6).

**Fig. 7:**
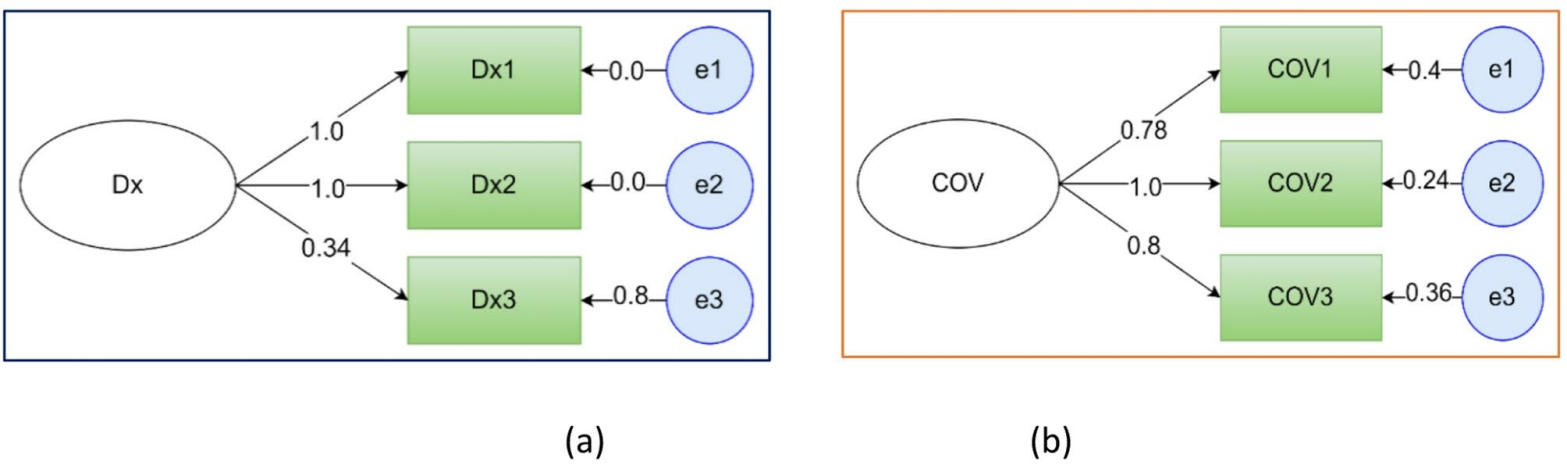
Factor loadings of history of malaria diagnosis and COVID-19 related factors observable variables. (**a** DX = diagnosis history observable factors, (**b**) cov = COVID-19 related observable factors

Similarly, history of malaria diagnosis was assessed as a latent variable by history of travel among the patients and their families as shown in the figure below. History of COVID 19 test and vaccination was also assessed as a latent variable using three observed variables as shown below (Fig. 7).

**Fig. 8:**
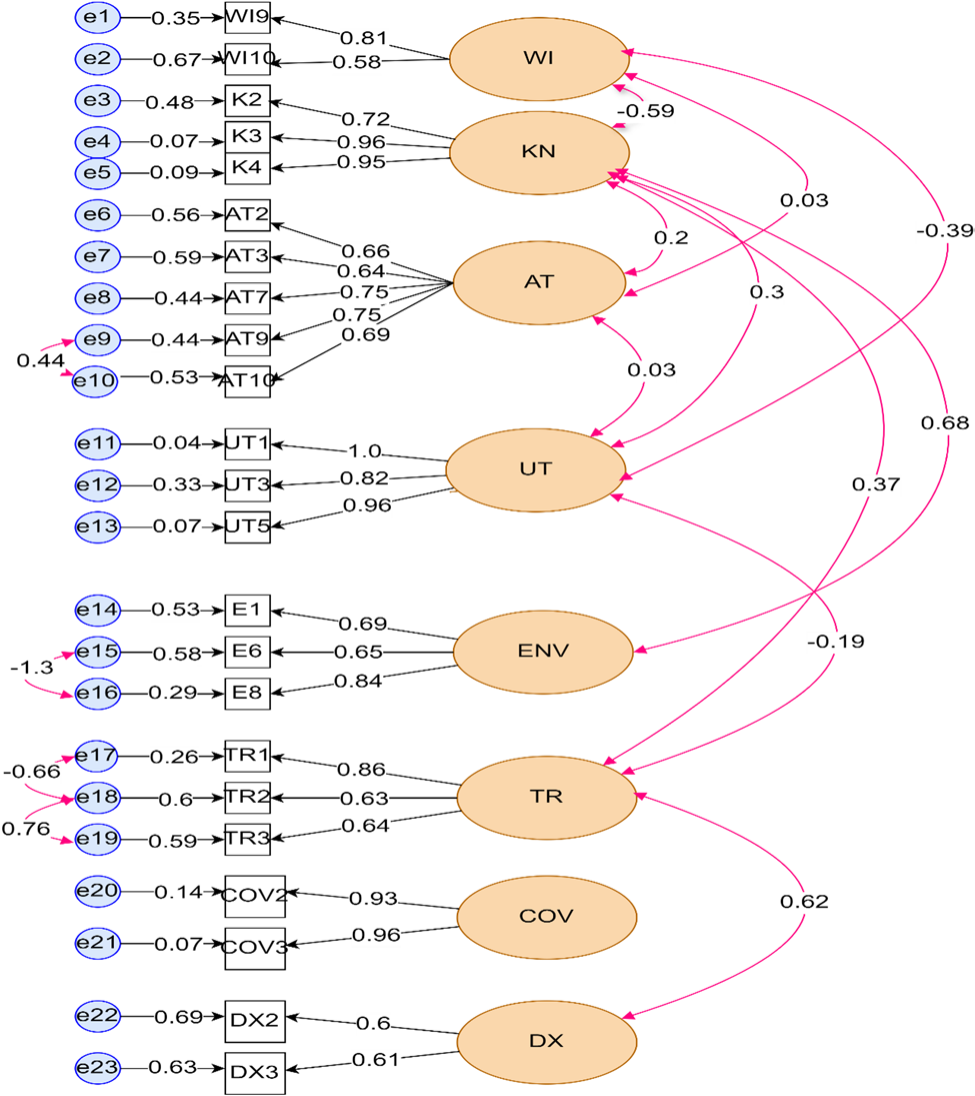
Standardized factor loading, variances and covariances of modified items from Confirmatory Analysis. Note: Visual representation of eight factor model for the malaria infection. Numbers to the left of the rectangles represent residuals (expressed as covariance). Numbers between the single-arrow-lines connecting latent variables and items indicate a factor loading values. Numbers between the bidirectional arrows connecting the latent variables imply a relationship between factors

The confirmatory factor analysis was done and those items with value above 0.5 were included in the final model. All those selected items were statistically significant (*p* < 0.05) (Table 2; Fig. 8). The measurement errors of both observed variables and latent variables was also calculated (S3).

**Table 2:** Standardized factor loadings of measurement variables in the final model

Latent Variables	Measurement variables	Standardized factor loading	*P*-value	Measurement errors
Wealth Index	WI9	0.81	< 0.001	0.347
	WI10	0.58	< 0.001	0.668
Knowledge	K2	0.72	< 0.001	0.48
	K3	0.96	< 0.001	0.07
	K4	0.95	< 0.001	0.09
Attitude	AT2	0.66	< 0.001	0.562
	AT3	0.64	< 0.001	0.586
	AT7	0.75	< 0.001	0.438
	AT9	0.75	< 0.001	0.44
	AT0	0.69	< 0.001	0.523
Utilization	UT1	1.0	< 0.001	−0.041
	UT3	0.82	< 0.001	0.331
	UT5	0.96	< 0.001	0.072
COVID-19	Cov2	0.93	< 0.001	0.143
	Cov3	0.96	< 0.001	0.07
Environment	E1	0.69	< 0.001	0.528
	E6	0.65	< 0.001	0.583
	E8	0.84	< 0.001	0.29
History of travel	TR1	0.86	< 0.001	0.261
	TR2	0.63	< 0.001	0.597
	TR3	0.64	< 0.001	0.587
History of malaria diagnosis	DX2	0.56	< 0.001	0.687
	DX3	0.61	< 0.001	0.632

### Final model

The cutoff values were adjusted based on our specific research context or theoretical considerations. We considered the common fit indices include: chi-square test (χ²- Lower values indicate better fit), comparative fit index (CFI- Values close to 1 indicate good fit (typically ≥ 0.95), root mean square error of approximation (RMSEA- Values ≤ 0.06 indicate good fit) and standardized root mean square residual (SRMR-Values ≤ 0.08 indicate good fit).

Accordingly, the values of those common modification indices values of the final model are as follows: chi-square test (χ²- 2.), comparative fit index (CFI- 0.988), tucker-lewis index (TLI-0.985); root mean square error of approximation (RMSEA- 0.051) and standardized root mean square residual (SRMR-0.071) (Table 3).

**Table 3:** Modification indices of the model

Model	X2	Df	X2/df	CFI	TLI	RMSEA	SRMR
Hypothetical			< 5	≥ 0.95	≥ 0.95	≤ 0.06	≤ 0.08
Modified model 1	534.1	189	2.82	0.964	0.956	0.075	0.128
Final model	447.1	222	2.01	0.988	0.985	0.051	0.071

*df* degree of freedom, *CFI* Comparative normed Fit Index, *TLI* Tucker-Lewis index, *RMSEA* Root Mean squared error of Approximation, *SRMR* Standardized Root Mean Square Residual

On the other hand, the item characteristics curve (ICC) of each latent variable for malaria infection was done. This helps for the interpretation of factor to item relationship and individual item score is on the x-axis and the conditional probability of item endorsement given the latent variables score is on the y-axis. Accordingly, items with steeper slopes have higher factor loadings than items with flatter slopes (Fig. 10).

**Fig. 9:**
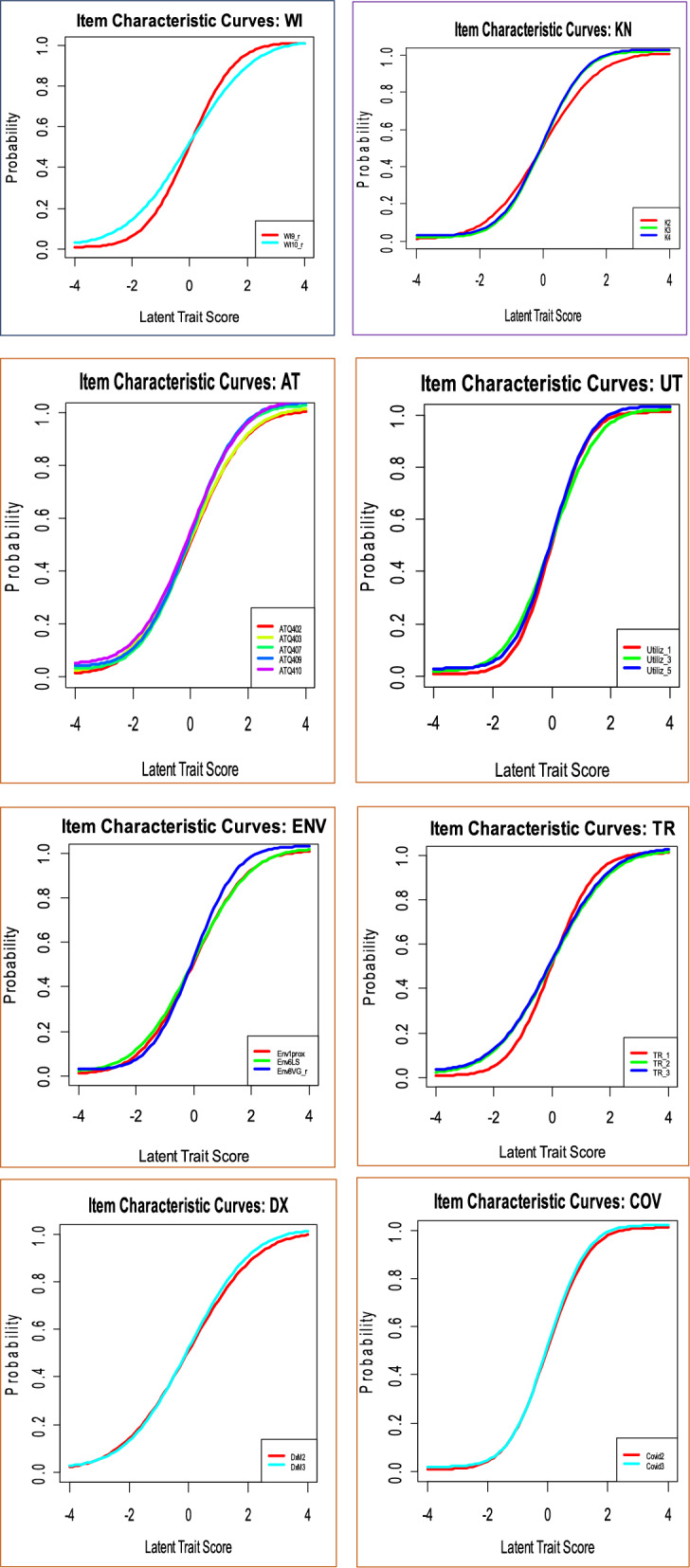
Item characteristics curve of latent variables

Wealth index had a negative statistically significant effect on ITN utilization (β = −0.66; *p* < 0.001), which means wealthier individuals or households are less likely to use ITNs compared to poorer ones. However, this study showed that both wealth Index and ITN utilization had no statistically significant effect on malaria infection (β = 0.232; *p* = 0.829; 0.18; *p* = 0.448). Similarly, wealth index had statistically significant negative effect on knowledge (β = −0.63; *p* < 0.001). The negative coefficient indicates that individuals or households with higher wealth tend to have lower knowledge levels compared to those with lower wealth. But, knowledge on malaria and its prevention in itself had no statistically significant effect on malaria infection (β = −0.12; *p* = 0.71). Attitude had positive effect on ITN utilization (β = 0.16; *p* = 0.049) which indicates more positive attitude is associated with increased use of ITNs. Having a history of travel outside the city had significant positive effect on malaria infection (β = 0.969; *p* = 0.01).

**Fig. 10:**
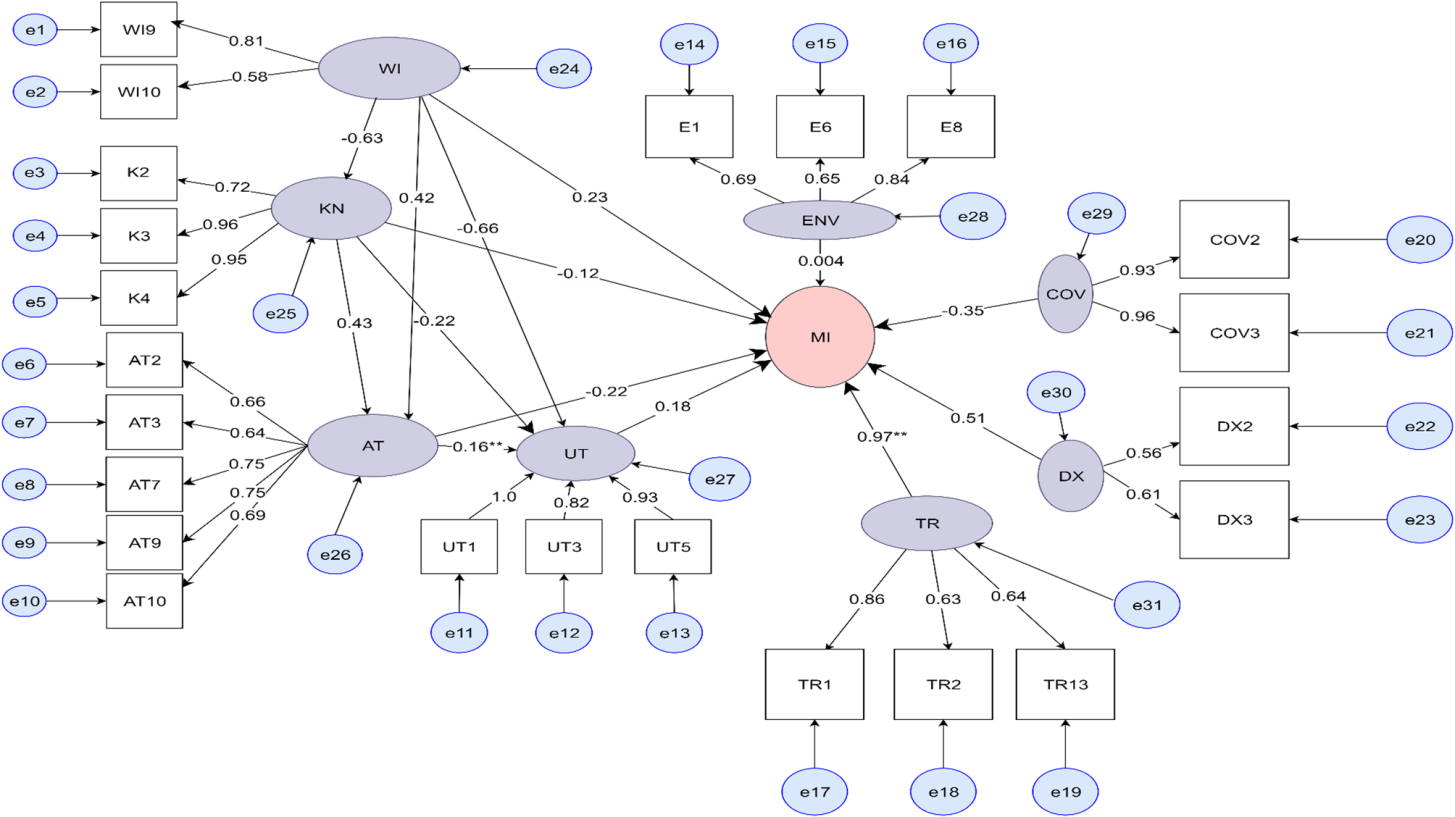
structural Equation Modeling testing the factors affecting malaria infection in Eastern Ethiopia. Note: The circles represent the latent variables and the rectangles represent observed variables. Numbers to the left of the rectangles represent residuals (expressed as covariance). Numbers between the single-arrow-lines connecting latent variables and items indicate a hypothesized direct effect (expressed as standardized regression coefficients)

The indirect effect analysis revealed two pathways in which attitude and ITN utilization as the mediating factor significantly influenced the malaria infection: [1] Wealth Index → Attitude → Malaria infection (indirect path coefficient β = −0.091; *p* = 0.038) [2] Attitude → Utilization → Malaria Infection (indirect path coefficient β = 0.029; *p* = 0.048) (Table 4) (Fig. 10).

**Table 4:** Estimates of structural model /both direct and indirect effects

Latent variables relationship	Standardized estimate	*P*-value
Direct effect		
WI → K	-0.634	<0.001**
WI → AT	0.423	0.413
K→ AT	0.43	0.577
K→ UT	-0.22	0.13
AT → UT	0.162	0.049**
WI → UT	0.66	<0.001**
WI → MI	0.232	0.829
K → MI	-0.12	0.705
AT → MI	-0.22	0.102
UT → MI	0.18	0.448
COV → MI	-0.346	0.396
E → MI	0.004	0.995
TR → MI	0.969	0.01**
DX → MI	0.514	0.303
Indirect effect		
Ind_WI_M_I__AT	-0.091	0.038**
Ind_WI_M_I__KN	0.073	0.706
Ind_WI_M_I__UT	-0.117	0.45
Ind_AT_M_I__UT	0.029	0.048**
Ind_KN_AT_M_I	0.059	0.548

** shows statistically significant at p value <0.05

(*Ind* Indirect effect, *MI* Malaria Infection, *WI* Wealth Index, *K *Knowledge, *AT *Attitude, *UT *Utilization, *E *Environment, *COV *COVID-19, *TR *Travel history, *Dx * history of diagnosis)

## Discussion

Malaria is one of the most important public health problems in low- and middle-income nations, and control and elimination needs more efforts due to the complex relationship of its risk factors mostly in endemic areas. This study used SEM, a new approach of modeling infectious diseases in Epidemiology, to assess the direct and indirect risk factors of malaria infection in urban setting of eastern Ethiopia where *An. stephensi* invasion was reported [47].

The results from the final model fitting analysis showed that all the selected observational variables were statistically significant, and the structural model was reasonably established. In this study, the sociodemographic index was not statistically significant and hence, was removed from the final model. Regarding the direct effect, wealth index was assessed and it had significant negative effect on ITN utilization which is consistent with finding from South Central Ethiopia [49], Ghana [50] and Mozambique [51]. However, it is inconsistent with finding from Demographic and Health survey data of 11 East African countries [52] and Southern Ethiopia [53] central Ethiopia [54]. This might be due to the fact that rich peoples may consider other prevention methods which may affect ITN utilization. Moreover, rich people may have good house structure which may protect them from indoor vector biting although they may still be exposed to outdoor biting mosquitoes in the early evening and morning hours when they spend outside their houses [53].

Similarly, wealth index had a negative effect on knowledge of malaria prevention and control. This is similar with classical epidemiologic study from Afar region, northeastern Ethiopia which showed individuals with low income were about three times more likely to have good knowledge about malaria and its prevention and control measures compared to those with high income [55]. This might be because rich people may have busy schedules with their business and may not have time to attend malaria prevention campaign and awareness provided at the health institutions and in the communities. The direct effect also showed that attitude had positive effect on ITN utilization, indicating that a more positive attitude or good perception was associated with a greater ITN utilization. The indirect effect analysis revealed that attitude and ITN utilization as the mediating factor significantly influenced the malaria infection. The finding from Northwest Ethiopia revealed that a high level of perceived barriers was negatively associated with ITN utilization, with individuals reporting higher perceived barriers being less likely to use ITNs compared to those with lower perceived barriers. The difference might be due to differences in the type of statistical analysis employed in those studies [56].

In this study, with a direct effect, a very strong positive relationship with a large, standardized estimate of travel history and malaria infection was identified. Though no statistically significant finding was report from North west Ethiopia [57], this is in line with the findings from South Western Ethiopia [20], Uganda [19], Guinea [58], Malawi [18] and systematic review from sub-Saharan Africa [59] that revealed the associated of malaria with travel history. This is essential to know whether people are being infected in the urban areas or during travel for better focus and designing of appropriate intervention [17]. The similarity might be due to increased risk of exposure in high-transmission areas and a decrease in protective behaviors when people are away from home.

However, environmental factors and history of malaria diagnosis had positive direct effect on malaria infection, but they were not statistically significant. Similarly, in this study, the self-response to COVID-19 test and vaccination status had no effect on malaria infection though a previous study found relation between them [60].

Structural equation modelling (SEM), which has long history in application in the area of psychology, ecology, social sciences, and econometrics, is gaining increasing application in infectious diseases research. Its application to study disease transmission remains limited, given that epidemiologists want to delineate causes and effects from observational data. Its use in recent studies, including epidemiologic studies, has proven valuable for exploring novel ideas particularly in understanding complex interconnections among observed and latent variables [31, 36, 47].

### Strength and limitation

One of the strengths of this study is the use of SEM using lavaan, the latest package of R software which is comprehensive and flexible for model specification and estimation. Moreover, utilizing DWLS as an estimation method is particularly advantageous for handling categorical and ordinal data and small sample sizes, enhancing the robustness and accuracy of the findings. On the other hand, the study has some limitations. There is a potential for recall bias, which may have been introduced by certain knowledge-based questions that are difficult to remember. Similarly, there might be information bias due to ITN utilization and COVID-19 tests, and vaccinations were self-reported. To minimize such biases, training on probing techniques has been given to the data collectors and supervisors. Another limitation of this study is lack of published article on the subject to compare these findings with specifically for the factors associated with the disease.

## Conclusion

In this study, SEM analysis revealed significant insights into the multifaceted dynamics influencing urban malaria infection. The findings demonstrated that several factors exerted direct effects on the likelihood of malaria infection, including wealth index, ITN utilization, knowledge, attitude, and history of travel. These factors underscore the complexity of malaria transmission in urban settings, where socio-economic, behavioral, and environmental determinants intersect. Notably, the study identified indirect pathways that further illuminate the nuanced relationships among these variables. The wealth index not only had a direct impact on malaria infection but also exerted an indirect effect mediated through attitude. This highlights how socio-economic status influences perceptions and behaviors, which in turn affect malaria risk. Similarly, attitude was found to indirectly influence malaria infection through its impact on ITN utilization, suggesting that fostering positive attitudes toward preventive measures can enhance their uptake and effectiveness. Hence, advanced modeling is crucial in predicting factors that could play role either directly or indirectly in sustaining malaria transmission in urban areas. Furthermore, the finding of this study suggests the need to strengthen holistic approach and urban-targeted malaria interventions to prevent and control further urban malaria infection and spread. It is recommended to conduct a community-based study that integrates ecological and entomological data to assess the impact of environmental factors and vector dynamics on disease transmission.

## Supplementary Information

Supplementary Material 1: S1. Description of measurement items

Supplementary Material 2: S2. reliability analysis of observed variables 

Supplementary Material 3: S3. Measurement errors of observed and latent variables

## Data Availability

Data is provided within the manuscript or supplementary information files.

## Abbreviations

DWLS: Diagonally Weighted Least Square
ICMER: International Center of Excellence for Malaria Research
ITN: Insecticide Treated Nets
Lavaan: Latent Variable Analysis
LLIN: Long-lasting insecticidal nets
PCA: Principal Component Analysis
SEM: Structural Equation Modeling
WHO: World Health Organization
