# Supplementary material for: Modeling urban malaria infection in Anopheles stephensi hotspot area in Eastern Ethiopia: application of Structural Equation Modeling
Source: BMC Infect Dis. 2025 Nov 5;25:1502. doi: 10.1186/s12879-025-11841-2 (PMC12587530; doi:10.1186/s12879-025-11841-2)
Supplement: Supplementary file 1 — Supplementary Material 1: S1. Description of measurement items [file 12879_2025_11841_MOESM1_ESM.docx]

**S1: Description of measurement items**

| **Constructs** | **Sub-constructs** | **Measured Items** |
| --- | --- | --- |
|  | Sociodemographic index | SD5ES: Educational status |
|  |  | SD6OS: Occupational status |
|  |  | SD7FS: Family size |
|  | Environmental factors | E1: Household proximity to the health facility/clinic |
|  |  | E2: Household distance from the water body |
|  |  | E3: Is stagnant water found around your home/area? |
|  |  | E4: Your home distance from the main road |
|  |  | E6: own any livestock |
|  |  | E7: Is water container in found in your compound |
|  |  | E8: Is there vegetation/agricultural land in your living compound? |
|  | Wealth Index | WI1: Number of rooms |
|  |  | WI2: Main material of the floor |
|  |  | WI3: Main material of the roof |
|  |  | WI4: Presence of eves and other holes on the wall of the house |
|  |  | WI5: house owner |
|  |  | WI6: Energy source to prepare food |
|  |  | WI7: Do you have a separate kitchen from the main house? |
|  |  | WI8: Main source of drinking water |
|  |  | WI9: What kind of toilet facility your family-owned? (reverse coded) |
|  |  | WI10: Do you share this toilet facility with other households? (reverse coded) |
|  |  | WI11: Where does your cooking take place? |
|  | Knowledge related factors | K1: Have you ever heard of an illness called malaria? |
|  |  | K2: Do you know mosquito-breeding sites? |
|  |  | K3: Do you think that this malaria vector affects urban population than rural population |
|  |  | K4: Do you think that nowadays malaria vectors can breed in any artificial water con |
|  |  | K5: Do you know how malaria is transmitted from person to person? |
|  |  | K6: In your opinion, which people are more affected by malaria in your area? |
|  |  | K7: Do you think that malaria prevention and control is possible? |
|  |  | K8: Do you know drugs to be prescribed for malaria patients? |
|  |  | K9: Did you buy malaria drug without physician prescription? |
|  | Attitude related factors | AT0: I think that Malaria is a serious and life-threatening (fatal) disease |
|  |  | AT1: I am sure that anyone can get Malaria |
|  |  | AT2: I think malaria is transmitted from person to person similar with other communicable diseases |
|  |  | AT3: I think the best way to prevent myself from getting Malaria is to avoid getting mosquito bite |
|  |  | AT4: I think that it is dangerous when Malaria medicine is not taken completely |
|  |  | AT5: I can buy anti-Malaria drugs from the drug shop/pharmacy to treat myself |
|  |  | AT6: I believe sleeping under a mosquito net during the night is one way to prevent malaria |
|  |  | AT7: I think malaria is greater risk for child, pregnant women, malnutrition person and migrants |
|  |  | AT8: I might be at a greater risk of getting Malaria if I work and sleep overnight |
|  |  | AT9: I think malaria is a curable disease |
|  |  | AT10: I think that I should go to the health facility to have my blood tested as soon as I suspect that I have suffered from Malaria |
|  |  | AT11: I might be at a greater risk of getting Malaria if I don’t fully cover all my body |
|  |  | AT12: I will seek for advice or treatment when I get Malaria |
|  | ITN utilization related factors | UT1: Is this household own of any bed net now |
|  |  | UT2: Has anyone in the household ever sold or given away a mosquito net |
|  |  | UT3: household member purchase the nets |
|  |  | UT4: Slept under ITN last night |
|  |  | UT5: Slept under ITN last week |
|  | History of travels outside the study area | TR1: thought travel outside the town is risk for malaria infection |
|  |  | TR2: Do you have history of travel outside this town within the last 2 weeks? |
|  |  | TR3: In the past one month, did you travel outside and or sleep overnight there? |
|  | History of malaria diagnosis | Dx1: thought history of malaria diagnosis is risk factor |
|  |  | Dx2: Have you been diagnosed for malaria before? |
|  |  | Dx3: Were any of your family members in your house diagnosed for malaria in the past |
|  | COVID-19 impact | Covid1: thought that COVID-19 has impact for malaria infection |
|  |  | Covid2: Received COVID-19 test |
|  |  | Covid3: Received COVID-19 vaccine |
