# Supplementary material for: Modeling urban malaria infection in Anopheles stephensi hotspot area in Eastern Ethiopia: application of Structural Equation Modeling
Source: BMC Infect Dis. 2025 Nov 5;25:1502. doi: 10.1186/s12879-025-11841-2 (PMC12587530; doi:10.1186/s12879-025-11841-2)
Supplement: Supplementary file 2 — Supplementary Material 2: S2. reliability analysis of observed variables [file 12879_2025_11841_MOESM2_ESM.docx]

**S2: reliability analysis of observed variables**

| **Environmental factors** | Reliability (Alpha) |
| --- | --- |
| E1 | 0.54 |
| E2 | 0.12 |
| E3 | 0.22 |
| E4 | 0.76 |
| E6 | 0.52 |
| E7 | 0.22 |
| E8 | 0.62 |
| **Wealth Index factors** |  |
| WI1 | 0.5304 |
| WI2 | 0.5654 |
| WI3 | 0.4204 |
| WI4 | 0.5425 |
| WI5 | 0.598 |
| WI6 | 0.5875 |
| WI7 | 0.5669 |
| WI8 | 0.5718 |
| WI9 | 0.4528 |
| WI10 | 0.522 |
| WI11 | 0.5667 |
| Knowledge related variables |  |
| K1 | 0.375 |
| K2 | 0.624 |
| K3 | 0.73 |
| K4 | 0.867 |
| K5 | 0.3258 |
| K6 | 0.619 |
| K7 | 0.3387 |
| K8 | 0.3774 |
| K9 | 0.39 |
| Attitude related variables |  |
| AT0 | 0.8301 |
| AT1 | 0.8187 |
| AT2 | 0.8218 |
| AT3 | 0.82 |
| AT4 | 0.8179 |
| AT5 | 0.8585 |
| AT6 | 0.8107 |
| AT7 | 0.8155 |
| AT8 | 0.8143 |
| AT9 | 0.8111 |
| AT10 | 0.8139 |
| AT11 | 0.8253 |
| AT12 | 0.8142 |
| Utilization related variables |  |
| UT1 | 0.7641 |
| UT2 | 0.8619 |
| UT3 | 0.8625 |
| UT4 | 0.7568 |
| UT5 | 0.7391 |
| Travel history |  |
| TR1 | 0.78 |
| TR2 | 0.69 |
| TR3 | 0.76 |
| History of malaria diagnosis |  |
| Dx1 | 0.55 |
| Dx2 | 0.71 |
| Dx3 | 0.57 |
| COVID 19 test and vaccine |  |
| COV1 | 0.81 |
| COV2 | 0.58 |
| COV3 | 0.78 |

**E= environmental, WI= Wealth Index, K=knowledge, AT= Attitude, UT= Utilization, TR=Travel history, DX=history of malaria diagnosis, COV= COVID19*
