# Supplementary material for: Modeling urban malaria infection in Anopheles stephensi hotspot area in Eastern Ethiopia: application of Structural Equation Modeling
Source: BMC Infect Dis. 2025 Nov 5;25:1502. doi: 10.1186/s12879-025-11841-2 (PMC12587530; doi:10.1186/s12879-025-11841-2)
Supplement: Supplementary file 3 — Supplementary Material 3: S3. Measurement errors of observed and latent variables [file 12879_2025_11841_MOESM3_ESM.docx]

**S3: Measurement errors of observed and latent variables**

| Measured variables | Measurement errors |
| --- | --- |
| WI9 | 0.347 |
| WI10 | 0.668 |
| K2 | 0.48 |
| K3 | 0.07 |
| K4 | 0.09 |
| AT2 | 0.562 |
| AT3 | 0.586 |
| AT7 | 0.438 |
| AT9 | 0.44 |
| AT10 | 0.523 |
| UT1 | -0.041 |
| UT3 | 0.331 |
| UT5 | 0.072 |
| Covid2 | 0.143 |
| Covid3 | 0.07 |
| E1 | 0.528 |
| E6 | 0.583 |
| E8 | 0.29 |
| TR1 | 0.261 |
| TR2 | 0.597 |
| TR3 | 0.587 |
| Dx2 | 0.687 |
| Dx3 | 0.632 |
| WI | 1.0 |
| K | 0.487 |
| AT | 0.784 |
| UT | 0.746 |
| COV | 1.0 |
| E | 1.0 |
| TR | 1.0 |
| DX | 1.0 |
